# Evaluation of the Immunohistochemical Scoring System of CDX2 Expression as a Prognostic Biomarker in Colon Cancer

**DOI:** 10.3390/diagnostics14101023

**Published:** 2024-05-15

**Authors:** Andreea-Corina Ilie-Petrov, Daniel-Alin Cristian, Florin Andrei Grama, Andrei Chitul, Angela Blajin, Andrei Popa, Draga-Maria Mandi, Luminița Welt, Marina Alina Bara, Rareș Vrîncianu, Carmen Maria Ardeleanu

**Affiliations:** 1 Faculty of Medicine, “Carol Davila” University of Medicine and Pharmacy, 020021 Bucharest, Romania; andreea.petrov@drd.umfcd.ro (A.-C.I.-P.); daniel.cristian@umfcd.ro (D.-A.C.); mandi.draga@gmail.com (D.-M.M.); cmardeleanu@yahoo.com (C.M.A.); 2 Clinical General Surgery Department, Colțea Clinical Hospital, 030171 Bucharest, Romania; angela.blajin@yahoo.com (A.B.); andrei.popa10@gmail.com (A.P.); 3 Pathology Department, Colțea Clinical Hospital, 030171 Bucharest, Romania; luminitawelt@gmail.com (L.W.); marina.bara08@gmail.com (M.A.B.); 4 Medical Oncology Department, Colțea Clinical Hospital, 030171 Bucharest, Romania; raressvrincianu@gmail.com; 5 Pathology Department, OncoTeam Diagnostic Laboratory, 010719 Bucharest, Romania

**Keywords:** colon cancer, biomarker, CDX2, differentiation grade, tumor budding score, immunohistochemistry, scoring system

## Abstract

Encoded by the CDX2 homeobox gene, the CDX2 protein assumes the role of a pivotal transcription factor localized within the nucleus of intestinal epithelial cells, orchestrating the delicate equilibrium of intestinal physiology while intricately guiding the precise development and differentiation of epithelial tissue. Emerging research has unveiled that positive immunohistochemical expression of this protein shows that the CDX2 gene exerts a potent suppressive impact on tumor advancement in colorectal cancer, impeding the proliferation and distant dissemination of tumor cells, while the inhibition or suppression of CDX2 frequently correlates with aggressive behavior in colorectal cancer. In this study, we conducted an immunohistochemical assessment of CDX2 expression on a cohort of 43 intraoperatively obtained tumor specimens from patients diagnosed with colon cancer at Colțea Clinical Hospital in Bucharest, between April 2019 and December 2023. Additionally, we shed light on the morphological diversity within colon tumors, uncovering varying differentiation grades within the same tumor, reflecting the variations in CDX2 expression as well as the genetic complexity underlying these tumors. Based on the findings, we developed an innovative immunohistochemical scoring system that addresses the heterogeneous nature of colon tumors. Comprehensive statistical analysis of CDX2 immunohistochemical expression unveiled significant correlations with known histopathological parameters such as tumor differentiation grades (*p*-value = 0.011) and tumor budding score (*p*-value = 0.002), providing intriguing insights into the complex involvement of the CDX2 gene in orchestrating tumor progression through modulation of differentiation processes, and highlighting its role in metastatic predisposition. The compelling correlation identified between CDX2 expression and conventional histopathological parameters emphasizes the prognostic significance of the CDX2 biomarker in colon cancer. Moreover, our novel immunohistochemical scoring system reveals a distinct subset of colon tumors exhibiting reserved prognostic outcomes, distinguished by their “mosaic” CDX2 expression pattern.

## 1. Introduction

According to the latest data from GLOBOCAN 2022, colorectal cancer (CRC) stands as the third leading cause of mortality globally, exhibiting notably elevated incidence and mortality rates across diverse regions, predominantly in Asia, Europe, and the USA, affecting individuals of all genders [1]. In Romania, CRC holds the second position among the foremost causes of mortality and occupies a leading position regarding the incidence across genders, particularly demonstrating a heightened prevalence among women [1]. Based on current statistical insights, CRC demonstrates a higher prevalence in economically developed nations with medium to high income levels, showcasing fluctuations in occurrence linked to lifestyle choices [1,2]. On the other hand, in developed nations, there is a significant decline in mortality rates attributed to enhanced patient accessibility to preventative and screening initiatives, alongside the adoption of personalized oncological treatments, marking a significant stride in combating the disease [3]. As per the latest 2022 guideline by the European Society for Medical Oncology (ESMO), an estimated 15–30% of patients are diagnosed at advanced disease stages with metastases frequently located in the liver, which notably yield a survival prognosis of less than 3 years, underscoring the urgency for enhanced therapeutic interventions [3,4].

CRC comprises a spectrum of profoundly heterogeneous tumors, characterized by a multifaceted pathogenesis driven by genetic and epigenetic changes that selectively affect tumor suppressor genes, oncogenes, and genes integral to DNA repair pathways [5,6]. The introduction of precision medicine marks a transformative shift in oncological practice, fostering a deeper comprehension of the intricate molecular and genetic mechanisms underpinning carcinogenesis, culminating in the identification of specific biomarkers endowed with diagnostic, predictive, and prognostic capabilities [5,7,8,9]. This advancement enables the optimization of treatment strategies customized to individual patients or molecular subgroups of CRC, demonstrating broad applicability across the spectrum of disease stages [5,7,8,9].

Our study provides a concise overview of the current state of molecular pathology in CRC, detailing the established and emerging molecular biomarkers that are shaping the management of this disease. By providing this background, we underscore the importance of integrating clinical, histopathological, and molecular data to fully grasp the complex and heterogeneous nature of CRC. This comprehensive approach not only advances our understanding but also sets the stage for our focused investigation into the caudal-related homeobox transcription factor (CDX2), a novel prognostic biomarker that has recently garnered attention but is not yet recognized in the current clinical guidelines of leading organizations such as ESMO and the National Comprehensive Cancer Network (NCCN) [3,8,10,11].

To this end, we conducted an immunohistochemical evaluation of CDX2 expression in 43 colon cancer cases to assess its prognostic capabilities further. Our analysis focused on correlating CDX2 expression with established clinical, histopathological, and molecular parameters. Concurrently, we developed a novel immunohistochemical scoring system that accommodates tumor heterogeneity and addresses the absence of standardized scoring criteria [9]. This initiative aims to lay the groundwork for a universally accepted scoring system for CDX2, thereby bolstering its prognostic significance within the scientific community. Additionally, our study sheds light on the morphological diversity within colon tumors, uncovering varying differentiation grades within the same tumor. These findings not only reflect variations in CDX2 expression but also highlight the genetic complexity underlying these tumors. Ultimately, our enhanced immunohistochemical scoring system is designed to improve the prognostic accuracy of CDX2, supporting its potential as a critical tool in the oncological management of CRC. Lastly, this study is part of a broader doctoral project led by A.-C.I.-P. as the PhD student and supervised by C.M.A., with the main goal of exploring several novel histopathological markers, including tumor microenvironment factors, and molecular biomarkers, such as CDX2 immunohistochemical expression, and how they relate to established clinical, histopathological, and molecular parameters, as well as patient outcomes, in order to understand their impact on CRC prognosis.

## 2. Molecular Pathology in Colorectal Cancer

The onset of colorectal carcinogenesis unfolds as an intricate journey, triggered by the disturbance of specific cellular signaling pathways, culminating in the disruption of cellular homeostasis [5]. The disruption arises from external oncogenic influences like lifestyle choices or internal factors such as intestinal inflammation, initiating a gradual buildup of genetic and epigenetic changes, ultimately triggering dysplasia in the intestinal tissue [5,12,13].

As outlined in the current literature, the onset of CRC unveils two predominant molecular pathways: the conventional adenoma–carcinoma route and the serrated alternative pathway, each originating from distinct precancerous lesions [5,13,14]. In these pathways, a cascade of distinct genetic mutations unfolds, including alterations in the APC gene, associated with conventional adenomatous polyps, and mutations in the BRAF gene, linked to serrated polyps [12,13,14,15]. Additionally, three carcinogenic mechanisms have been described, with chromosomal instability (CIN) being the most common, frequently implicated in the adenoma–carcinoma molecular pathway [13]. This mechanism is marked by a plethora of chromosomal irregularities stemming from mutations in key suppressor genes such as APC, SMAD4, and TP53, as well as mutations in the oncogene KRAS, which intricately interact with the Wnt and MAPK signaling pathways [5,12,13,14,15]. The second mechanism involves the Mismatch Repair (MMR) system and is commonly linked with the serrated molecular pathway [12,13]. The MMR system consists of four proteins (MLH1, PMS2, MSH2, and MSH6) responsible for correcting genetic abnormalities occurring during DNA replication, such as those encountered in chromosomal microsatellite regions [12,13,14,16,17]. When a mutation arises in one of the genes responsible for synthesizing the four MMR proteins, the system undergoes inefficiency (dMMR), frequently via epigenetic mechanisms, resulting in the accumulation of errors like insertion–deletion loops or mispairing of DNA bases, a phenomenon termed high microsatellite instability (MSI-H) [5,12,13,14]. Due to the expression of mutational neoantigens, the dMMR/MSI-H phenotype frequently associates with a vigorous immune response marked by an abundance of tumor-infiltrating lymphocytes (TILs) [12,15,18,19]. The third mechanism involves the hypermethylation of genetic promoter regions rich in CpG islands (CIMP) and is commonly associated with the serrated pathway, along with mutations in the APC, MLH1, and/or CDX2 genes [4,12,14,20,21]. Recent studies reveal a significant association between the CIMP mechanism and mutations in the BRAF gene lacking CpG islands in the promoter region, a phenomenon that still requires further research to be fully understood; nonetheless, this association hints at a plausible involvement of these mutations in instigating or perpetuating this mechanism [12,18,22].

The intricate interplay of genetic and epigenetic mechanisms, coupled with the elaborate network of molecular pathways implicated in colorectal carcinogenesis, underscores the diverse morphological and molecular landscape of these tumors. As a result, precise oncologic management requires thorough examination of histopathological and molecular parameters to facilitate precise decision-making concerning both prognosis and treatment strategies for CRC.

## 3. Exploring Molecular Biomarkers in Colorectal Cancer

In the context of managing CRC, an integrated approach combines clinical, paraclinical, and histopathological parameters to effectively categorize CRC into distinct stages utilizing the TNM (Tumor Node Metastasis) classification system pioneered by the American Joint Committee on Cancer (AJCC), thereby facilitating both prognostic evaluation and the development of targeted therapeutic interventions [3,7,10,11,23]. While TNM staging remains the cornerstone in oncological patient care, its effectiveness is limited by the inability to account for genetic mutations and molecular alterations in CRC, which are crucial for accurate prognosis and therapeutic decisions [7,23]. Incorporating genetic and molecular biomarker testing into CRC management has improved the TNM staging system, offering a more comprehensive understanding of disease dynamics, fostering the development of personalized therapeutic approaches, and enabling a more precise evaluation of prognosis and treatment effectiveness [7,23]. For example, insights into MMR/MSI status or mutations in the RAS (NRAS, KRAS) and BRAF genes can guide personalized therapies and enhance prognosis predictions, as highlighted below.

In CRC, genetic and molecular biomarkers play a pivotal role across all stages, furnishing vital insights into potential genetic susceptibility to cancer (e.g., Lynch syndrome or familial adenomatous polyposis), disease prognosis (risk of recurrence or tumor progression), and/or therapeutic effectiveness [24]. Among the essential biomarkers in CRC management, seamlessly integrated into routine medical practice as per the latest ESMO and NCCN guidelines, lies the assessment of MMR/MSI status, offering valuable insights into prognosis, therapeutic efficacy, and genetic susceptibility to cancer such as Lynch syndrome [10,11,15]. In advanced CRC, the evaluation of RAS genes’ (NRAS, KRAS) mutational status is recommended for predicting therapeutic response, while the assessment of the BRAF gene’s mutational status is conducted for prognostic purposes [3,10,15].

Currently, there is an unprecedented surge in the identification of clinically and therapeutically relevant molecular targets, with numerous novel biomarkers emerging, offering significant potential in guiding therapeutic decisions and prognostic evaluation. These include immunohistochemical analysis of CDX2 expression for prognostic stratification, tumor stroma examination for prognostic assessment, and TILs’ evaluation for both prognostic and predictive purposes, alongside ongoing research exploring genetic profiling with prognostic implications in advanced CRC, and genotyping through liquid biopsy-derived circulating tumor DNA (ctDNA) or circulating tumor cells (CTCs) [11,23,25,26,27,28].

## 4. Unraveling the Impact of the CDX2 Gene on Colorectal Cancer

CDX2 is a protein expressed in the nuclei of intestinal epithelial cells and is encoded by the CDX2 homeobox gene [7,9]. The caudal-related homeobox gene family, which encompasses CDX1, CDX2, and CDX4, forms an essential component of the ParaHox gene cluster, exerting a critical influence on the embryonic development of the digestive tract [9]. While CDX1 and CDX4 contribute to embryogenesis—CDX1 in intestinal differentiation and CDX4 in axial elongation—CDX2 plays a crucial role in intestinal cell specification and differentiation [9]. Its absence results in major abnormalities in intestinal structure and function [9]. CDX2 is commonly expressed in colorectal cancers, making it a valuable marker for understanding the disease [9]. In contrast, CDX1 and CDX4 are typically not evaluated in CRC due to their less significant roles in adult intestinal function [9]. Regarding the distribution of CDX2 gene expression, it begins in the duodenum, surging gradually to peak intensity in the epithelium of the distal small intestine (ileum) and proximal colon, while being entirely absent in the distal rectum, where the CDX1 gene dominates [9,29,30].

CDX2 assumes a crucial function in maintaining intestinal equilibrium, participating in the development and differentiation of epithelial tissue through the activation of specific intestinal genes, such as mucin 2 (MUC2), sucrase–isomaltase, and carbonic anhydrase I (CA1), while also impeding epithelial proliferation by halting the cell cycle when DNA is damaged [4,9,30,31]. Furthermore, by modulating intra- and intercellular trafficking through the regulation of RAB11A and KIF3B, CDX2 maintains the integrity of the intestinal epithelium; RAB11A facilitates intracellular endosome recycling, while KIF3B supports vesicle distribution essential for cell communication, thus preventing inflammatory intestinal conditions [9]. In vivo and in vitro studies have revealed that the lack of CDX2 gene expression results in macrophage accumulation within the intestinal mucosa [9,29,31]. Last but not least, emerging findings suggest that the CDX2 gene acts as a tumor suppressor, hindering tumor cell growth and spread by interfering with the Wnt/β-catenin signaling pathway [4,7,31,32,33,34]. High expression of the CDX2 gene in CRC is linked to a 50% lower death rate compared to cases where it is low or absent, and its proper functioning enhances survival rates without disease progression or tumor recurrence [7,35]. Recent research suggests that the impairment of CDX2 gene function is linked to epigenetic mechanisms like CIMP, involving hypermethylation of its CpG island-rich promoter region [4,7,9,21,34,36].

### 4.1. Deciphering the Relationship between the CDX2 and Clinicopathological Parameters Alongside Molecular Biomarkers

In multiple instances, researchers have demonstrated that diminished or absent CDX2 expression is associated with the aggressive behavior of CRC, characterized by invasive and poorly differentiated tumors, as well as low overall survival (OS) and disease-free progression (DFS) rates [7,9,21,31,34,36,37,38].

Although researchers have shown that there is no statistically significant correlation between the age and sex of patients and tumors exhibiting reduced or absent CDX2 gene expression, some studies have suggested a greater prevalence of CDX2-negative tumors in elderly women [34,36,37,39,40]. Concerning the primary tumor site, research suggests that tumors exhibiting diminished or negative CDX2 expression are commonly found in the right colon, including the cecum, ascending colon, and transverse colon [9,32,34,40,41].

Colorectal tumors exhibiting diminished or negative CDX2 expression display aggressive behavior, as indicated by Xu et al.’s study, which highlights a statistically significant correlation between the degree of CDX2 gene dysfunction and the level of tumor invasion [4]. This observation is corroborated by additional research, including the study conducted by Issac et al., which validates that negative CDX2 expression was commonly detected in invasive pT3 (confined to the serosa) and pT4 (exceeding the serosa) tumors [32,40,42]. Studies have also highlighted that CDX2 expression correlates with the degrees of differentiation and histological subtypes, indicating that poorly differentiated tumors often exhibit diminished or negative CDX2 expression and are commonly mucinous in nature [31,34,36,40]. Last but not least, there is a minor discrepancy among studies: Dawson et al. identify a direct correlation between CDX2 loss and perineural invasion, while El Rafeay et al. find a less consistent association with perineural but a clear inverse correlation with lymphovascular and locoregional lymph node invasion [42,43]. Asgari-Karchekani et al. further note a strong link between reduced CDX2 expression and extensive locoregional lymph node involvement [34]. Despite these specific differences, the overall findings from these researchers converge on the conclusion that CDX2-negative tumors frequently exhibit forms of aggressive invasion, consistently emphasizing the marker’s predictive value regarding tumor aggressiveness [34,42,43].

Tumoral budding, characterized by small clusters of detached tumor cells located at the invasion front or within the tumor microenvironment, represents a morphological manifestation of a broader phenomenon known as epithelial–mesenchymal transition (EMT) [31,44]. This process facilitates the mobility of tumor cells and contributes to cancer progression by promoting migration and invasion into surrounding tissues [44]. Tumor budding is associated with an unfavorable prognosis when tumors exhibit a high number of peri- and intratumoral buds [31,44]. Recent studies indicate that CDX2-negative tumors are frequently associated with the fourth category of the Consensus Molecular Subtype Classification (CMS) of CRC developed around 2015 by an international consortium of researchers [9,45]. This category is associated with genetic mutations that sustain the EMT process, thereby accelerating the tumor invasion process [12]. Studies suggest that inhibiting the CDX2 gene activates the EMT process by loss of junctional proteins [31,32]. Additionally, Hansen et al. highlighted in their study that tumor buds exhibit negative CDX2 expression [46].

The mutation of the BRAF gene in advanced CRC typically forecasts an unfavorable prognosis; however, instances have been observed where outcomes are unexpectedly favorable despite this mutation [47]. Recent research, such as the findings presented by Aasebø et al., reveal that in some BRAF-mutated CRCs, elevated CDX2 expression is associated with significantly better prognoses [47]. This improved outlook is primarily due to CDX2’s role as a tumor suppressor gene, which in these cases, mitigates the typically adverse impacts of the BRAF mutation [47]. Alternatively, when both the CDX2 and BRAF genes are affected—CDX2 primarily through downregulation of expression and BRAF through mutation—they typically indicate an unfavorable prognosis [4,9,36,47]. Regarding the RAS gene family, researchers have not identified any statistically significant correlation between the CDX2 expression and the mutational status of the KRAS and NRAS genes [36,48].

### 4.2. Evaluation of CDX2 Expression in Colorectal Cancer

The CDX2 protein demonstrates robust nuclear expression within non-neoplastic intestinal epithelial cells and is reliably identified in the vast majority of CRC histological subtypes (90–100%), including metastases originating from colorectal tissue, thereby functioning as an immunohistochemical marker for confirming or excluding intestinal origin in secondary tumors of unknown origin [9,31,34,36,40]. However, in 10–30% of CRC cases, a reduced or negative CDX2 expression has been observed [7,37,40,49]. The CDX2 protein, while commonly associated with colorectal tumor cells, exhibits expression in various tumors of the digestive system, including duodenal (100%), gastric (71%), and pancreatic (30%) tumors, and may additionally be detected in neuroendocrine tumors and mucin-producing malignancies like bladder, ovarian, pulmonary, and biliary carcinomas [9,34,39].

The clinical utility of immunohistochemical results is strictly dependent on the methods employed for assessing immunoreactivity, including largely standardized scoring methods [50]. 

In CRC, the immunohistochemical evaluation of newly identified biomarkers relies on threshold-based scoring methods, which are arbitrarily set and vary between studies, lacking precise reproducibility, resulting in semi-quantitative and conflicting results across similar studies [50,51,52]. 

Three notable constraints of modern immunohistochemistry have been recognized, comprising a limited scoring spectrum (typically an ordinal intensity scale ranging from 1, weak, to 3, strong), human error (manifesting as coloration artifacts), and the lack of result reproducibility [51]. Besides the ordinal scoring system, certain pathologists employ a complex scoring method known as the immunoreactive score (IRS), which incorporates the classical intensity score multiplied by the proportion of positive tumor cells, thereby providing an indication of the intensity level [51].

Recent research has introduced diverse strategies to tackle this challenge, including the utilization of digital analysis as a dependable substitute for traditional scoring techniques, although necessitating precise calibration of the employed platform due to the array of algorithms involved [53]. An alternative approach involves employing the Receiver Operating Characteristic (ROC) curve alongside the bootstrapping replication method, facilitating the validation of immunohistochemical findings derived from the cut-off scoring method and ensuring their dependable reproducibility [50]. Artificial intelligence (AI) has recently become integrated into medical practice, demonstrating utility across various medical specialties including radiology, surgery, forensic medicine, and even pathology. AI programs function as an alternative and adjunctive tool for modern histopathology; however, they demand more intricate oversight from pathologists in terms of training the AI system (machine learning) to identify patterns, thus adding complexity to the implementation of this technology [52]. Digital pathology using AI technology aids in overcoming the aforementioned limitations by automating scoring systems in immunohistochemistry, thus mitigating human subjectivity [52].

Given that CDX2 is a recently identified biomarker, its clinical efficacy is still being explored, and the immunohistochemical scoring methods utilized to evaluate its expression in CRC employ cut-off criteria, resulting in semi-quantitative findings with varying expression percentages and discrepancies regarding its correlation with other clinicopathological and molecular parameters, as indicated by studies investigating this biomarker [9].

After reviewing recent studies regarding the assessment of CDX2 expression in CRC, various immunohistochemical scoring models utilizing cut-off thresholds have been identified as follows:

(1) Model 1 (separate assessment of the number of stained cells and staining intensity): (a) score for stained cells: 0 (0%), 1 (>0–25%), 2 (>25–50%), 3 (>50–75%), and 4 (>75%), and (b) staining intensity score: 0 (negative), 1 (weak positive), and 3 (strong positive) [4,36].

(2) Model 2 (separate assessment of the number of stained cells and staining intensity): (a) score for stained cells: 0 (<5%), 1+ (5–25%), 2+ (26–50%), 3+ (51–75%), and 4+ (>75%), and (b) staining intensity score: focal staining (<50%) and diffuse staining (>50%) [20,54].

(3) Model 3 (common assessment of the number of stained cells and staining intensity): negative score for unstained or weakly stained cells (<10%), low score (+1) for weak to moderate staining (10–29% of cells), intermediate score (+2) for moderate to strong staining (30–49% of cells), and high score (+3) for strong staining (>50% of cells) [34].

(4) Model 4 (separate assessment of the number of stained cells and staining intensity): (a) a four-tiered scoring system for staining intensity: 1 (negative), 2 (weak), 3 (moderate), and 4 (strong), and (b) score for stained cells: 1 (0–1%), 2 (2–10%), 3 (11–25%), 4 (26–50%), 5 (51–75%), and 6 (>75%) [47].

(5) Model 5 (common assessment of the number of stained cells and staining intensity): score A: unstained tissue (0–5% positive cells), score B: weak or scattered staining in a minority of cells (5–49% positive cells), score C: moderate or strong staining in a majority of cells (50–59% positive cells), and score D: strong staining in all cells (95–100% positive cells) [45].

(6) Model 6 comprised of two groups of staining (CDX2^neg^ and CDX2^pos^) each further divided into two subgroups: (a) CDX2neg group: score 0 (negative CDX2 expression) and score 0.5 (weak or scattered nuclear expression in a minority of cells), and (b) CDX2pos group: score 2 (strong staining in a majority of cells) and score 3 (strong staining in all cells) [31,38].

The variety of cut-off models employed in studies investigating CDX2 expression in CRC highlights the pressing requirement for the development of a standardized scoring system aimed at objectifying immunohistochemical results [9,52]. A standardized scoring system will enable the integration of immunohistochemical evaluation of CDX2 expression into clinical practice and will also facilitate comparative studies of published data regarding this biomarker [9,52].

Another crucial consideration highlighted in the study conducted by Badia-Ramentol et al. is that the majority of scoring models utilized to assess CDX2 expression in CRC fail to consider tumor heterogeneity, simplistically categorizing tumors as either CDX2-positive, CDX2-moderately/weakly stained, or CDX2-negative based solely on the proportion of stained cells, despite the potential presence of heterogeneous staining intensity patterns within the tumor, which could impact prognosis [9]. In our study, we aim to analyze this CDX2 expression diversity among different cancer clones within the same tumor, and to devise a new scoring system that encapsulates this intratumoral heterogeneity.

## 5. Materials and Methods

### 5.1. Case Selection—Inclusion and Exclusion Criteria

In our study, we carefully outlined inclusion criteria to focus on cases of colon cancer where elective surgery was performed to remove the primary tumor, with no immunohistochemical assessment of CDX2 expression conducted on the excised tumors. Additionally, we ensured that all participants had not undergone previous oncological treatment before surgery, as prior treatment could potentially alter tumor conditions.

Considering the significant correlation in the scientific literature between moderate or negative CDX2 expression and invasive colon tumors, we selectively included pT3 and pT4 histopathologically staged colon tumor specimens. This aimed to optimize the likelihood of identifying the moderate and/or negative CDX2 expression pattern and higher invasive potential of colon tumors. Our study strategically focused on these advanced disease stages to emphasize findings relevant to aggressive tumor behaviors. Originally, our dataset included a broader range of stages; however, to ensure the specificity and relevance of our results to aggressive tumor behaviors, we meticulously selected only those cases most critical for our research objectives. This targeted approach was not due to sample exclusion errors but was a deliberate methodological choice to enhance the clinical applicability of our novel scoring system. By concentrating on these particular stages, our research provides essential insights into the prognostic implications of CDX2 in the progression of invasive colon cancers.

Exclusion criteria involved rectal cases to maintain a homogenous study group of colon tumors. This decision was driven by the significant differences in treatment and tumor biology between colon and rectal cancers. Rectal cancer often involves preoperative radiation, unlike colon cancer, which can alter tumor conditions and CDX2 expression profiles. By focusing solely on colon tumors, particularly those of pT3 and pT4, we aimed to minimize confounding factors introduced by varied treatments associated with rectal cases, thereby ensuring clearer insights into CDX2 expression and its prognostic implications specific to colon cancer management.

Overall, our study provides valuable insights into CDX2 expression and its prognostic relevance in colon cancer, particularly at advanced disease stages (stages II, III, and IV). Additionally, it is noteworthy that all included cases happened to have clear margins of resection, contributing to a more comprehensive representation of tumor characteristics within our sample.

### 5.2. Histopathological and Immunohistochemical Assessment

This study was carried out at the Pathology Department of the OncoTeam Diagnostic Laboratory in Bucharest, utilizing a sample of 43 colon tumor specimens collected from patients who underwent colon cancer surgery at Colțea Clinical Hospital in Bucharest between April 2019 and December 2023. General informed consent was provided by the patients upon hospital admission. This retrospective study was approved by the Institutional Review Board (Ethics Committee of Colțea Clinical Hospital) under protocol number 35 dated 21 February 2024.

The selection of the 43 cases was conducted based on the predefined inclusion and exclusion criteria through the examination of medical records spanning the period from 2019 to 2023, retrieved from the archives of the Pathology Department at Colțea Clinical Hospital in Bucharest. Furthermore, additional data was extracted from these records for comprehensive analysis, encompassing demographic parameters (gender and age), clinical features (oncological disease stage, colon tumor location), histopathological characteristics (stenotic tumor nature, extent of tumor invasion, histological subtype, tumor budding score), and molecular aspects (MMR/MSI status and mutational status of the KRAS gene). 

To immunohistochemically evaluate CDX2 expression in the 43 colon cancer cases selected for this study, a collaborative effort was undertaken with the Pathology Department of Colțea Clinical Hospital in Bucharest. The collaboration facilitated the identification and retrieval of formalin-fixed paraffin-embedded (FFPE) colon tumor samples from specimens excised during surgeries conducted between April 2019 and December 2023. Promptly after the surgery and in adherence to the department’s established internal protocols, these samples were processed to prepare the hematoxylin and eosin (H&E)-stained slides used to provide a diagnosis at that moment in time. Both H&E slides and the corresponding FFPE tumor sample were meticulously stored during the last 5 years, under controlled cool and dry conditions, within the Pathology Department of Colțea Clinical Hospital to preserve tissue integrity.

All H&E-stained slides pertaining to the 43 cases, retrieved from the archive, were analyzed microscopically by A.-C.I.-P. and two experienced pathologists from the Pathology Department (L.W. and M.A.B.) in order to identify the FFPE sample that encompassed adequate tumor tissue and adjacent normal colonic mucosa. Notably, the inclusion of normal tissue—specifically from resection margins histologically confirmed as free of tumor involvement—was critical for qualitative immunohistochemical analysis of CDX2 expression. For each of the 43 cases, one H&E-stained slide along with its corresponding FFPE tumor sample containing sufficient tumor tissue and adjacent normal colonic mucosa was selected for detailed study.

Immunohistochemical staining was conducted at the OncoTeam Diagnostic Laboratory in Bucharest, using the 43 FFPE tumor samples retrieved from the archive of the Pathology Department of Colțea Clinical Hospital. The staining process adhered to the laboratory’s established internal protocols. The FFPE tumor samples were sliced into 2 μm thick sections and subsequently stained with BOND™ Ready-To-Use CDX2 Primary Antibody (clone EP25, Leica Biosystems Newcastle Ltd., Newcastle Upon Tyne, UK), which was selected based on the laboratory’s available inventory of antibodies. The application of the antibody was facilitated by the BOND-III Fully Automated Immunostaining System (Leica Biosystems, Wetzlar, Germany), ensuring consistency and reproducibility in the immunostaining process.

Two examiners, including C.M.A., an experienced pathologist and PhD supervisor, and A.-C.I.-P., a PhD student and surgeon in training with relevant experience in pathology derived from her specialization in forensic pathology, conducted the histopathological and immunohistochemical evaluation. They meticulously examined under the microscope, the 43 H&E-stained slides retrieved from the Pathology Department of Colțea Clinical Hospital, as well as the 43 CDX2-stained slides at the Pathology Department of the OncoTeam Diagnostic Laboratory in Bucharest. The process involved an initial assessment by the PhD student, followed by a comprehensive verification by the PhD supervisor, ensuring a thorough and precise analysis of the results.

On the 43 H&E-stained slides, the two examiners meticulously analyzed the heterogeneity of differentiation within the tumors, noting each grade of differentiation observed and quantifying its proportion relative to the total tumor area. This evaluation was performed at 20× magnification, which is commonly used for detailed analysis of tissue architecture and cellular differentiation. The histopathological characteristics of the colon tumor evaluated on the H&E-stained slides—such as extent of tumor invasion, histological subtype, and tumor budding score—were retrieved from the archived reports and taken into consideration as additional data in the statistical study.

On the 43 CDX2-stained slides, the normal colonic mucosa was used as a positive internal reference in the analysis of the immunohistochemical expression intensity level of CDX2 at the tumor level. Additionally, only the nuclear expression of CDX2 in tumor cells was taken under consideration. The decision to focus solely on the nuclear expression of CDX2 stems from its role as a nuclear transcription factor, which is critical in the regulation of gene expression within intestinal epithelial cells. CDX2 expression evaluation involved assessing both the intensity of nuclear staining and the percentage of stained nuclei, carried out at 40x magnification to accurately observe nuclear details and ensure precise quantification of staining intensity.

The study aimed to assess the heterogeneity of CDX2 expression by specifically analyzing the range of nuclear CDX2 intensities within each evaluated tumor: moderate (lightly stained nuclei), positive (intensely stained nuclei), and negative (unstained nuclei). Additionally, the ratio of stained (positive and moderate intensity) and unstained nuclei was quantified relative to the total tumor area. This evaluation model takes into consideration each level of CDX2 intensity and proportion of stained and unstained nuclei observed within each evaluated tumor, distinguishing it from other models outlined in the previous Section 4.2 (Evaluation of CDX2 Expression in Colorectal Cancer). Such insights are crucial for the development of the newly proposed immunohistochemical scoring system detailed in the subsequent Section 6.1 (Development of a Novel Immunohistochemical Scoring System for Assessing CDX2 Expression in Colon Cancer).

The findings from the immunohistochemical analysis provided semi-quantitative data due to the inherent nature of immunohistochemical techniques, which often offer semi-quantitative rather than fully quantitative results.

### 5.3. Statistical Analysis

A descriptive statistical analysis was performed using the IBM^®^ SPSS^®^ Statistics version 29.0.2.0 (IBM Corp., Armonk, NY, USA) platform on the variables derived from both the evaluation of medical documentation corresponding to the selected cases and the immunohistochemical assessment of CDX2 expression.

The variables extracted from medical records encompass both numeric parameters like age, and categorical attributes such as patient gender (female, male), oncological staging (II, III, and IV), colon tumor location (right-sided tumor—cecum, ascending colon, transverse colon; left-sided tumor—descending colon, sigmoid colon), presence or absence of tumor stenosis, histological subtype (conventional adenocarcinoma; mucinous adenocarcinoma), tumor budding score (BD1—low score, 1–4 buds; BD2—moderate score, 5–9 buds; BD3—high score, >10 buds), tumor differentiation grade (G1—well-differentiated tumor; G2—moderately differentiated tumor; G3—poorly differentiated tumor), MMR/MSI status, and KRAS gene mutational status (KRAS-wildtype, KRAS-mutated). The CDX2 immunohistochemical expression results were subjected to statistical analysis as categorical variables.

The statistical analysis examined the association between the variables concerning CDX2 immunohistochemical assessment and those derived from medical records, encompassing clinical, histopathological, and molecular parameters. Fisher’s Exact, Chi-Squared, and Kruskal–Wallis tests were employed for this purpose, with statistical significance defined as a *p*-value below 0.05 for the entire dataset.

## 6. Results

### 6.1. Development of a Novel Immunohistochemical Scoring System for Assessing CDX2 Expression in Colon Cancer

As a key regulator of intestinal differentiation, CDX2 plays a central role in epithelial identity. Its expression levels are subject to modulation, leading to diverse patterns of expression intensities, thereby reflecting intratumoral variability [9]. Existing CDX2 cut-off immunohistochemical scoring models, as discussed in Section 4.2 (Evaluation of CDX2 Expression in Colorectal Cancer), tend to oversimplify by uniformly classifying tumors as CDX2-positive, -moderate, or -negative. This approach fails to capture the intratumoral variations and overlooks the significant implications that varying CDX2 intensity levels within the same tumor can have on clinical outcomes, thus undermining the accuracy of prognostic assessments. To address these limitations, we developed a new scoring system that accounts for the variability in the intensity level of CDX2 expression at the tumor level. Upon examining the 43 colon tumors included in this study, we observed three recurrent patterns of diverse CDX2 nuclear expression intensity. These observations led to the identification of three categories that reflect the heterogeneity of CDX2 expression within a tumor: Category 1, which exhibited positive and moderate CDX2 expression; Category 2, characterized by negative and moderate CDX2 expression; and Category 3, termed “mosaic”, which encompassed a combination of positive, moderate, and negative CDX2 expressions. Besides evaluating the intensity pattern of CDX2 in each of the 43 colon tumors analyzed in this study, we also measured the proportion of regions containing CDX2-stained (positive and moderate) and unstained nuclei relative to the total tumor area. This analysis was essential for further exploring Category 3 of CDX2 expression.

Additionally, by acknowledging the genetic and molecular heterogeneity apparent in colon tumors, we opted to devise a novel classification system centered on the varying degrees of tumor differentiation grades identified within each analyzed colon tumor, as observed on the H&E-stained slides. This endeavor aimed to explore the potential correlation between the diversity of CDX2 immunohistochemical expression and the variability in tumor differentiation observed within each analyzed case, as outlined in the forthcoming Section 6.2 (Descriptive Statistical Analysis) and Section 6.3 (Statistical Analysis of the Relationship between CDX2 Expression Categories and Clinicopathological and Molecular Parameters). Thus, four categories delineating tumor differentiation patterns emerged from the 43 analyzed colon cancer cases: (1) Category 1, which included grades G1 (well-differentiated) and G2 (moderately differentiated); (2) Category 2, composed solely of grade G2; (3) Category 3, termed “mosaic,” encompassing grades G1, G2, and G3 (poorly differentiated); and (4) Category 4, consisting of grades G2 and G3.

Based on this innovative immunohistochemical scoring method, aligned with the current literature indicating that moderate or negative CDX2 expression is associated with an unfavorable prognosis, while positive expression signals a good prognosis, we can classify Category 1 as indicative of a potential favorable prognosis and Category 2 as suggestive of a potential unfavorable prognosis. Regarding Category 3, identified in this study by a “mosaic” CDX2 expression pattern, prognostication may be deemed as reserved (variable).

### 6.2. Descriptive Statistical Analysis

Out of the 43 analyzed samples, 19 pertain to female patients (44.2%), and 24 pertain to male patients (55.8%). The mean age at diagnosis was 63.84 ± 1.521 years (ranging from 32 to 82 years). Female patients had an average age of 63.58 ± 2.573 years (ranging from 38 to 82 years), while male patients had an average age of 64.04 ± 1.860 years (ranging from 32 to 77 years). These demographic parameters are summarized in Table 1 for improved accessibility and clarity, aiding researchers and readers in their understanding of the analyzed data.

Colon tumors exhibit a higher frequency on the left side (55.8%) in contrast to the right side (44.2%). In females, left-sided colon tumor prevalence is notably higher (63.2%), whereas in males, tumor localization proportions are evenly distributed. Regarding oncological staging, 51.2% (*n* = 22) of cases were diagnosed at stage II, followed by stage III with a prevalence of 34.9% (*n* = 15), and stage IV with a prevalence of 14% (*n* = 6). Out of all the cases examined, 62.8% (*n* = 27) of the tumors exhibited stenosis. Moreover, an increased incidence of stenosing tumors was noted in stages III (73.3%) and IV (83.3%). Tumor invasion was confined to the serosa (pT3) in 90.7% of cases, while those extending beyond the serosa (pT4) accounted for 9.3%. Concerning the histological subtype of colon tumors, 72.1% (*n* = 31) were classified as conventional adenocarcinomas, while 27.9% (*n* = 12) were mucinous adenocarcinomas. Last but not least, the majority of colon cancer cases exhibited a moderate score of tumor budding (53.5%), while a minority presented a high score of tumor budding (20.9%). These clinical and histopathological parameters are summarized in Table 2 for improved accessibility and clarity, aiding researchers and readers in their understanding of the analyzed data.

Regarding the categories of tumor differentiation patterns observed in each analyzed colon cancer case, the predominant one was Category 1 (58.1%), followed by Categories 2 and 4, each demonstrating a similar prevalence (18.6%). Category 3, indicative of a “mosaic” pattern, was identified in a minority of cases, accounting for 4.7% of the total cohort (Table 3).

In the context of molecular evaluation, MMR/MSI status determination was conducted in 32 out of the 43 cases included in the study, while analysis of KRAS gene mutational status was performed in 21 out of the 43 cases. Among the total of 32 cases assessed for MMR/MSI status, 67.4% (*n* = 29) demonstrated a pMMR/MSS (efficient MMR system/stable microsatellite regions) phenotype, with only 7% (*n* = 3) displaying a dMMR/MSI-H phenotype. The three cases with a dMMR/MSI-H phenotype were linked to tumors diagnosed at oncological stage II. Out of the total 21 cases evaluated for KRAS gene mutational status, 25.6% (*n* = 11) showed KRAS gene mutation, while 23.3% (*n* = 10) had a wildtype status. Among the six cases diagnosed at oncological stage IV, only one tumor exhibited a wildtype status of the KRAS gene. These molecular parameters are summarized in Table 4 for improved accessibility and clarity, aiding researchers and readers in their understanding of the analyzed data.

Among the 43 cases examined immunohistochemically for CDX2 nuclear expression, the most commonly observed category was Category 3, characterized by “mosaic” CDX2 expression, accounting for 65.1% (*n* = 28), followed by Category 1, associated with a potentially favorable prognosis, comprising 23.3% (*n* = 10), and Category 2, associated with a potentially unfavorable prognosis, representing 11.6% (*n* = 5). The results regarding the three categories of CDX2 immunohistochemical expression are summarized in Table 5 for improved accessibility and clarity, aiding researchers and readers in their understanding of the analyzed data.

Category 3 of CDX2 nuclear expression was divided into four subcategories based on the proportion of CDX2-positive and CDX2-negative nuclei: (1) Subcategory 3A, featuring the highest percentage of CDX2-positive nuclei, succeeded by moderately stained CDX2 nuclei and then CDX2-negative nuclei (CDX2^(+)^ > CDX2^(±)^ > CDX2^(−)^); (2) Subcategory 3B, exhibiting the highest percentage of moderately stained CDX2 nuclei, followed by CDX2-positive nuclei and then CDX2-negative nuclei (CDX2^(±)^ > CDX2^(+)^ > CDX2^(−)^); (3) Subcategory 3C, displaying the highest percentage of CDX2-positive nuclei, trailed by CDX2-negative nuclei and then moderately stained CDX2 nuclei (CDX2^(+)^ > CDX2^(−)^ > CDX2^(±)^); and (4) Subcategory 3D, showcasing the highest percentage of moderately stained CDX2 nuclei, followed by CDX2-negative nuclei and then CDX2-positive nuclei (CDX2^(±)^ > CDX2^(−)^ > CDX2^(+)^). This subclassification was devised for analytical purposes, aiming to evaluate the correlation between the subcategories derived from Category 3 of CDX2 nuclear expression and the categories of tumor differentiation patterns, outlined in the next Section 6.3 (Statistical Analysis of the Relationship between CDX2 Expression Categories and Clinicopathological and Molecular Parameters). Among the total tumors exhibiting Category 3 of CDX2 expression (“mosaic” pattern), Subcategory 3A emerges as the most prevalent, constituting 32.6% (*n* = 14) of all cases examined, while Subcategory 3B follows closely with 23.3% (*n* = 10). Subcategories 3C and 3D exhibit comparable frequencies, each accounting for 4.7% (*n* = 2) of the total cases. The results regarding the subcategories derived from Category 3 of CDX2 nuclear expression are summarized in Table 6 for improved accessibility and clarity, aiding researchers and readers in their understanding of the analyzed data.

### 6.3. Statistical Analysis of the Relationship between CDX2 Expression Categories and Clinicopathological and Molecular Parameters

After conducting the Kruskal–Wallis test, there was no statistically significant association found between the categories of CDX2 immunohistochemical expression and the patient’s age at diagnosis (*p*-value = 0.146). Likewise, the Fisher’s Exact test did not identify any statistically significant correlation between the categories of CDX2 immunohistochemical expression and variables such as patient gender, colon tumor location, oncological stage, tumor invasion grade, stenosing tumor nature, and histological tumor subtype (Table 7).

The Fisher’s Exact test revealed a statistically significant association between the CDX2 immunohistochemical expression categories and the tumor budding score (*p* = 0.002) (Table 8).

Utilizing the Fisher’s Exact test, we analyzed the correlation between the CDX2 expression categories and the categories of tumor differentiation patterns, uncovering a statistically significant correlation (*p*-value = 0.011) (Table 9).

**Figure 1:**
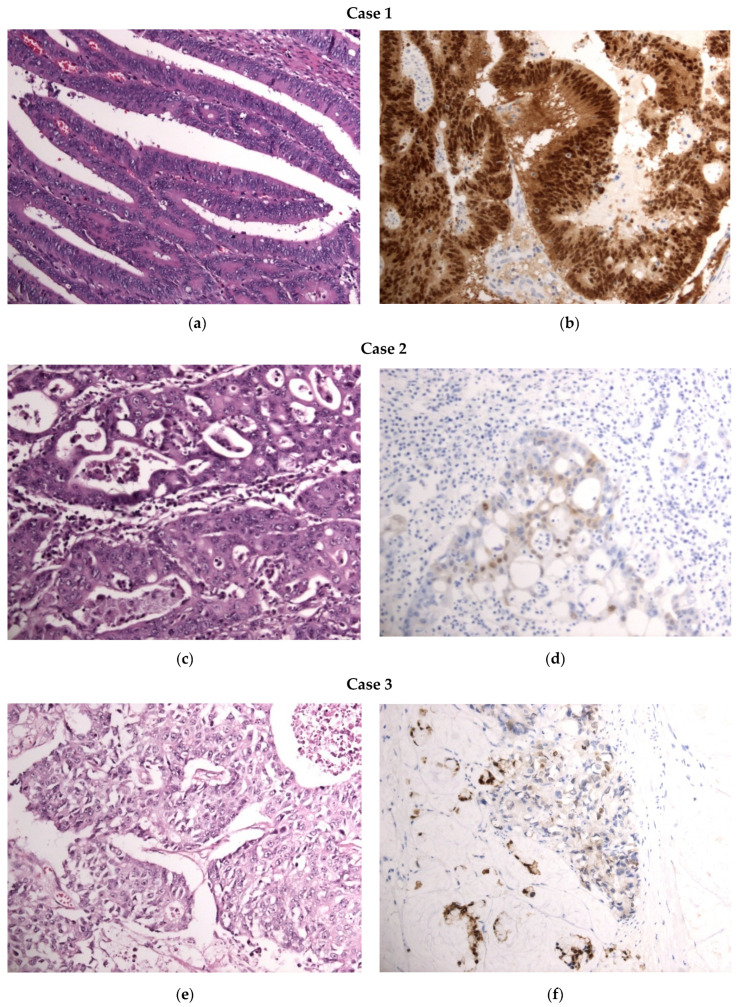
Illustration of the correlation between tumor differentiation patterns (H&E-stained slides on the left column, captured at 20× magnification) and CDX2 immunohistochemical expression categories (CDX2-stained slides on the right column, captured at 20× magnification) in a series of three colon tumors: Case 1: (**a**) Category 1 of tumor differentiation—well-differentiated (G1) and moderate differentiation (G2), (**b**) CDX2 expression Category 1—positive and moderate staining; Case 2: (**c**) Category 4 of tumor differentiation—moderate (G2) and poor differentiation (G3), (**d**) CDX2 expression Category 2—negative and moderate staining; Case 3: (**e**) Category 4 of tumor differentiation—moderate (G2) and poor differentiation (G3), (**f**) CDX2 expression Category 3, (“mosaic” pattern)—negative, moderate, and positive staining.

Our analysis further indicates that Category 1 of CDX2 expression, characterized by positive and moderate nuclear staining, is predominantly associated with well-differentiated (G1) and moderately differentiated (G2) colon tumors. Conversely, Category 2, marked by negative and moderate nuclear staining, frequently correlates with moderately and poorly differentiated (G3) tumors. Category 3, which encompasses positive, moderate, and negative staining of the nuclei, shows a variable association with all three grades of tumor differentiation. These observations are illustrated in the subsequent figure, which highlights three cases selected from our cohort that exemplify these associations (Figure 1).

Utilizing the Chi-squared test, we also analyzed the correlation between the subcategories derived from Category 3 of CDX2 nuclear expression and the categories of tumor differentiation patterns, uncovering a statistically significant correlation (*p*-value = 0.0018) (Table 10).

The use of statistical tests to assess potential correlations between molecular parameters —specifically MMR/MSI status and KRAS gene mutational status— and the categories of CDX2 expression in colon cancer is hindered by inconsistencies in the evaluation of these biomarkers across all oncological stages. In Romania, testing for MMR/MSI status is advised during oncological stages II and, under specific circumstances, stage III, whereas evaluation of KRAS gene mutational status is typically recommended solely for the oncological stage IV.

In relation to the status of the MMR/MSI biomarker, it is notable that among the tumors diagnosed at stage II, three out of 22 cases exhibited the dMMR/MSI-H phenotype. Of these instances, two are associated with the second CDX2 nuclear expression category, while the third case correlates with the third CDX2 nuclear expression category, characterized by a CDX2-negative nuclei percentage of 10%. Moreover, these three instances with the dMMR/MSI-H phenotype demonstrate a pronounced tumor budding score (tumor budding scores 2 and 3) and exhibit categories of tumor differentiation patterns 2 (G2) and 4 (G2, G3) (Table 11).

Regarding the statistical investigation into the relationship between KRAS gene mutational status and CDX2 immunohistochemical expression categories, no statistically significant association was observed between these variables (*p*-value = 0.298, as determined by the Fisher’s Exact test).

## 7. Discussions

Following our immunohistochemical evaluation of CDX2 expression in 43 cases of colon tumors and subsequent statistical analysis to investigate the relationship between immunohistochemical scoring categories and demographic variables like age and gender, our findings coincide with the research conducted by Çalık et al., which found no notable correlation between CDX2 expression and age or gender [37]. This concordance with our observations persists despite studies, such as the one conducted by Baba et al., indicating that negative CDX2 expression is predominantly observed in elderly women [39]. Additionally, in line with the research of Çalık et al. and Baba et al., we did not find any statistical correlation between CDX2 expression and tumor location, although some studies suggest that negative CDX2 expression is more commonly found in right-sided tumors [9,32,34,37,39,40,41].

Regarding the relationship between CDX2 expression and histological subtypes of CRC, the study conducted by Asgari-Karchekani et al. noted that mucinous adenocarcinoma typically exhibits negative or reduced CDX2 expression, in contrast to conventional adenocarcinoma, where positive CDX2 expression is more prevalent [34]. Similarly, Baba et al. observed that variations in CDX2 expression clearly delineate different histological subtypes, underscoring the biomarker’s role in distinguishing between mucinous and non-mucinous forms of CRC [39]. Conversely, studies such as those performed by Çalık et al. and Issac et al., along with our own findings on the 43 analyzed cases of colon cancer, did not observe significant correlations between CDX2 expression and the histological subtypes of CRC, emphasizing the inconsistency and potential subtype-specificity of CDX2 expression across different studies [37,40].

Our statistical analysis also investigated the correlation between CDX2 expression categories and clinicopathological parameters such as oncological stage and tumor invasion grades, and found no significant correlations. The specific inclusion criteria of our study, which focused exclusively on colon cancer cases histopathologically staged as pT3 and pT4, may explain this absence of correlation. This decision of including higher-grade tumors, as explained in Section 5.1 (Case Selection—Inclusion and Exclusion Criteria), was influenced by research such as that by Issac et al. and Xu et al., which identified a significant link between negative CDX2 expression and higher invasion grades (pT3 and pT4) [4,40]. Consistent with these studies, our analysis revealed that a majority of the analyzed colon cancer cases (33 out of 43) exhibited negative CDX2 expression, falling into categories 2 and 3 of CDX2 expression. Regarding the relationship between CDX2 expression and the stenosing nature of colon tumors, our analysis did not find a significant correlation. Although the stenosing characteristic does not directly measure tumor size, it suggests substantial growth that impairs organ function by blocking normal passage. Research like Baba et al.’s indicates that reduced CDX2 expression correlates with larger tumor sizes, suggesting that CDX2 levels may reflect tumor growth dynamics in CRC [39]. In contrast, Çalık et al.’s study, similar to ours, found no significant correlation between CDX2 expression and this parameter [37].

Our study evaluates the impact of CDX2 expression on colon cancer pathology, noting that most immunohistochemical scoring system models may not fully capture tumor heterogeneity. These models typically categorize CDX2 expression simply as positive, moderately/weakly stained, or negative, based solely on the percentages of stained or unstained nuclei. However, it is crucial to acknowledge that distinct regions within a tumor may exhibit varying intensities of CDX2 staining, potentially exerting a profound impact on prognosis and therapeutic outcomes [9]. In recognition of these limitations, we devised an innovative immunohistochemical scoring system for CDX2 expression assessment (CDX2 expression categories 1, 2, and 3), as well as a novel classification of the tumor differentiation patterns (categories 1, 2, 3, and 4), shedding light on the complex molecular heterogeneity of colon cancer.

Through our statistical analysis, we unveiled noteworthy correlations between the novel CDX2 immunohistochemical expression categories, based on patterns of CDX2 intensity, and conventional histopathological parameters used to assess tumor behavior in colon cancer, including tumor budding score and the newly introduced tumor differentiation categories. These findings resonate with current research which suggests that inhibition of the CDX2 gene triggers the process of EMT, morphologically represented by the phenomenon of tumor budding [1,12,31,32,44]. Additionally, they align with studies highlighting the tumor-suppressive role of the CDX2 gene, which substantially impacts tumor differentiation, correlating moderate or negative CDX2 expression with tumors exhibiting moderate to poor differentiation [4,32,33,34,40].

Furthermore, our study illustrates that tumors exhibit a range of differentiation patterns rather than a single dominant grade, based on the most prevalent type within the tumor. It also underscores the variability in tumor differentiation grades within colon tumors, indicative of the heterogeneity in CDX2 expression; specifically, it emphasizes that variations in CDX2 intensity are associated with a spectrum of distinct tumor differentiation grades. Our analysis reveals that Category 1, characterized by positive and moderate nuclear staining, is commonly associated with well- and moderately differentiated tumors (G1, G2). In contrast, Category 2, marked by negative and moderate nuclear staining, often correlates with moderately and poorly differentiated tumors (G2, G3). For a deeper analysis, Category 3, which represents a “mosaic” pattern of CDX2 expression, was further subdivided into subcategories 3A, 3B, 3C, and 3D, based on the varying percentages of CDX2 intensity. This detailed classification revealed that Subcategories 3A and 3B, which show higher percentages of CDX2-positive and moderate nuclei, significantly correlate with well- and moderately differentiated grades (G1, G2). Conversely, Subcategory 3C, with higher percentages of moderate to negative CDX2 nuclei, and Subcategory 3D, with a mixture of high percentages of both positive and moderate nuclei, frequently associate with a “mosaic” pattern of differentiation covering all grades (G1, G2, and G3) for the former, and moderate and poor differentiation (G2, G3) for the latter. This comprehensive approach allows us to better understand the intricate role of CDX2 in colon cancer differentiation.

Regarding the association between the categories of CDX2 expression and the conventional biomarkers, such as the mutational status of the KRAS gene and the status of the MMR/MSI system, the statistical analysis faced limitations due to the irregular assessment of these markers across all stages of colon cancer. In Romania, evaluating MMR/MSI status is mainly advised for oncological stages II and III, while assessing the mutational status of the KRAS gene is specifically recommended for stage IV colon cancer.

## 8. Conclusions

In conclusion, our study has introduced a refined immunohistochemical scoring system for CDX2 expression that considers the inherent heterogeneity of colon tumors—a detail often missed by conventional immunohistochemical scoring systems. This innovation has enabled us to uncover significant correlations between CDX2 expression patterns and key histopathological parameters, such as tumor budding and differentiation grades, which are instrumental in assessing tumor behavior, thus bolstering the prognostic significance of the CDX2 biomarker in colon cancer.

Our novel immunohistochemical scoring system, alongside the categorization of tumor differentiation patterns, has not only revealed a significant link between the diversity of CDX2 expression and the heterogeneity of colon cancer differentiation but also provided a new lens through which to view the evaluation of colon tumors. By meticulously analyzing the “mosaic” CDX2 expression category, we have highlighted a unique subgroup of tumors that could exhibit a variable prognosis. Additionally, this study underscores the importance of an integrated assessment of both histopathological and molecular characteristics of colon tumors to accurately predicting a prognosis.

These insights enhance our comprehension of CDX2’s role in colon cancer and support its integration into routine prognostic assessments as a dependable biomarker. They also highlight the imperative need for a standardized immunohistochemical scoring system. To build on this foundation, future studies should explore the longitudinal impact of CDX2 expression patterns on patient outcomes, ideally through multicentric studies, to validate and potentially standardize our novel scoring system across different clinical settings. This would not only bolster the clinical utility of CDX2 but also facilitate the development of new personalized therapeutic strategies for colon cancer patients.

## 9. Limitations

This study presents several methodological limitations that warrant consideration. It utilizes a semi-quantitative approach for the immunohistochemical assessment of CDX2 expression, which, while informative, lacks the precision of fully quantitative methods. Furthermore, the study is conducted within a single center and involves a relatively small patient cohort, which may restrict the external validity and generalizability of the findings to broader populations. The research focuses exclusively on colon tumors, deliberately excluding rectal cancers to maintain homogeneity in the sample, given the biological and treatment differences between colon and rectal cancers. This exclusion limits the comprehensive applicability of the results across all colorectal cancers. Additionally, the inclusion criteria favoring tumors with higher invasion grades (pT3 and pT4) may not accurately reflect the behavior and prognostic implications of CDX2 expression in earlier stages of colon cancer. The absence of a standardized immunohistochemical scoring system further complicates the reproducibility and comparison of findings across different studies and clinical settings. Moreover, the recent study period (2019–2023) constrains the analysis regarding long-term outcomes and other critical oncological follow-up parameters, thereby limiting insights into the longitudinal impact of CDX2 expression on patient prognosis. These factors collectively underscore the need for further research to refine the assessment methods of CDX2 and expand the scope of investigation to enhance the prognostic utility of CDX2 in colorectal cancer management.

## Figures and Tables

**Table 1:** Descriptive statistical analysis of the demographic parameters.

Gender	*n* (%)	Mean Age (Years)	Standard Error of the Mean (SEM) (Years)	Minimum Age (Years)	Maximum Age (Years)
Both genders	43 (100%)	63.84	1.521	32	82
Females	19 (44.2%)	63.58	2.573	38	82
Males	24 (55.8%)	64.04	1.860	32	77

**Table 2:** Descriptive statistical analysis of the clinical parameters (colon tumor location and oncological stage) and histopathological parameters (stenotic tumor nature, tumor invasion grade, histological tumor subtype, and tumor budding score).

Total Number of Cases Analyzed (*n*) = 43		*n*-Value	Percentage (%)
Colon tumor location	Left side	24	55.8%
	Right side	19	44.2%
Oncological stage	Stage II	22	51.2%
	Stage III	15	34.9%
	Stage IV	6	14%
Stenotic tumor nature	Present	27	62.8%
	Absent	16	37.2%
Tumor invasion grade	pT3 grade	39	90.7%
	pT4 grade	4	9.3%
Histological tumor subtype	ADK NOS	31	72.1%
	ADK Mucinous	12	27.9%
Tumor budding score	BD1	11	25.6%
	BD2	23	53.5%
	BD3	9	20.9%

pT3: tumor confined to the serosa; pT4: tumor extending beyond the serosa; ADK NOS: adenocarcinoma not otherwise specified (conventional); ADK Mucinous: Mucinous adenocarcinoma; BD1: tumor budding score 1 (low score); BD2: tumor budding score 2 (moderate score); BD3: tumor budding score 3 (high score).

**Table 3:** Descriptive analysis of the categories of tumor differentiation patterns.

Total Number of Cases Analyzed (*n*) = 43		*n*-Value	Percentage (%)
Categories of tumor differentiation patterns	Category 1 (G1 and G2)	25	58.1%
	Category 2 (G2)	8	18.6%
	Category 3 (G1, G2 and G3)	2	4.7%
	Category 4 (G2 and G3)	8	18.6%

G1: well-differentiated tumor; G2: moderately differentiated tumor; G3: poorly differentiated tumor.

**Table 4:** Descriptive statistical analysis of the molecular parameters (MMR/MSI biomarker and KRAS gene biomarker) in relation to the oncological stages of the disease.

Total Number of Cases Analyzed (*n*) = 43			*n*-Value	Percentage (%)
MMR/MSI biomarker	Cases Evaluated for MMR/MSI Status		32	74.42%
	pMMR/MSS phenotype	Total Cases	29	67.4%
		Stage II	19	65.52%
		Stage III	9	31.03%
		Stage IV	1	3.45%
	dMMR/MSI-H phenotype	Total Cases	3	7%
		Stage II	3	100%
KRAS gene biomarker	Cases Evaluated for KRAS Gene Mutation		21	48.84%
	Presence of KRAS gene mutation	Total Cases	11	25.6%
		Stage II	4	36.36%
		Stage III	2	18.18%
		Stage IV	5	45.45%
	Wildtype status of KRAS gene	Total Cases	10	23.3%
		Stage II	2	20%
		Stage III	7	70%
		Stage IV	1	10%

MMR: Mismatch Repair; MSI: Microsatellite Instability; pMMR: Efficient Mismatch Repair; MSS: Microsatellite Stable; dMMR: Deficient Mismatch Repair; MSI-H: High Microsatellite Instability.

**Table 5:** Descriptive statistical analysis of the CDX2 immunohistochemical expression categories.

Total Number of Cases Analyzed (*n*) = 43		*n*-Value	Percentage (%)
CDX2 nuclear expression	Category 1 (positive and moderate expression of CDX2)	10	23.3%
	Category 2 (negative and moderate expression of CDX2)	5	11.6%
	Category 3 (moderate, positive, and negative expression of CDX2)	28	65.1%

**Table 6:** Descriptive analysis of the subcategories derived from Category 3 of CDX2 nuclear expression.

Total Number of Cases Analyzed (*n*) = 28		*n*-Value	Percentage (%)
Category 3 of CDX2 nuclear expression	Subcategory 3A (CDX2^(+)^ > CDX2^(±)^ > CDX2^(−)^)	14	32.6%
	Subcategory 3B (CDX2^(±)^ > CDX2^(+)^ > CDX2^(−)^)	10	23.3%
	Subcategory 3C (CDX2^(+)^ > CDX2^(−)^ > CDX2^(±)^)	2	4.7%
	Subcategory 3D (CDX2^(±)^ > CDX2^(−)^ > CDX2^(+)^)	2	4.7%

^(+)^: positive stained nuclei; ^(−)^: moderately stained nuclei; ^(±)^: negative (unstained) nuclei.

**Table 7:** The relationship between CDX2 immunohistochemical expression categories and demographic, clinical, and histopathological parameters.

Total Number of Cases Analyzed (*n*) = 43		*n* (%)	CDX2 Immunohistochemical Expression Categories			Fisher’s Exact Test (*p*-Value)
			Category 1 *n* (%)	Category 2 *n* (%)	Category 3 *n* (%)	
Sex	Males	24 (55.8%)	6 (25%)	3 (12.5%)	15 (62.5%)	1.000
	Females	19 (44.2%)	4 (21.1%)	2 (10.5%)	13 (68.4%)	
Colon tumor location	Left side	24 (55.8%)	8 (33.3%)	2 (8.3%)	14 (58.3%)	0.221
	Right side	19 (44.2%)	2 (10.5%)	23 (15.8%)	14 (73.7%)	
Oncological stage	Stage II	22 (51.2%)	6 (27.3%)	2 (9.1%)	14 (63.6%)	1.000
	Stage III	15 (34.9%)	3 (20%)	2 (13.3%)	10 (66.7%)	
	Stage IV	6 (14%)	1 (16.7%)	1 (16.7%)	4 (66.7%)	
Tumor invasion grade	pT3 grade	39 (90.7%)	8 (20.5%)	5 (12.8%)	26 (66.7%)	0.415
	pT4 grade	4 (9.3%)	2 (50%)	N/A	2 (50%)	
Stenosing tumor nature	Present	27 (62.8%)	4 (14.8%)	4 (14.8%)	19 (70.4%)	0.254
	Absent	16 (37.2%)	6 (37.5%)	1 (6.3%)	9 (56.3%)	
Histological tumor subtype	ADK NOS	31 (72.1%)	5 (16.1%)	5 (16.1%)	21 (67.7%)	0.122
	ADK Mucinous	12 (27.9%)	5 (41.7%)	N/A	7 (58.3%)	
**Age**	All genders	43 (100%)	10 (23.3%)	5 (11.6%)	28 (65.1%)	Kruskal–Wallis’s test (*p*-value = 0.146)

pT3: tumor confined to the serosa; pT4: tumor extending beyond the serosa; N/A: Not Available; ADK NOS: adenocarcinoma not otherwise specified (conventional); ADK Mucinous: Mucinous adenocarcinoma.

**Table 8:** The relationship between CDX2 immunohistochemical expression categories and tumor budding scores.

Total Number of Cases Analyzed (*n*) = 43		*n* (%)	CDX2 Immunohistochemical Expression Categories			Fisher’s Exact Test (*p*-Value)
			Category 1 *n* (%)	Category 2 *n* (%)	Category 3 *n* (%)	
Tumor budding score	BD1	11 (25.6%)	6 (54.5%)	N/A	5 (45.5%)	0.002 *
	BD2	23 (53.5%)	4 (17.4%)	1 (4.3%)	18 (78.3%)	
	BD3	9 (20.9%)	4 (44.4%)	N/A	5 (55.6%)	

BD1: tumor budding score 1 (low score); BD2: tumor budding score 2 (moderate score); BD3: tumor budding score 3 (high score); N/A: Not Available. * A *p*-value < 0.05 is considered statistically significant.

**Table 9:** The relationship between the categories of CDX2 nuclear expression and Tumor differentiation patterns.

Total Number of Cases Analyzed (*n*) = 43		*n* (%)	CDX2 Immunohistochemical Expression Categories			Fisher’s Exact Test (*p*-Value)
			Category 1 *n* (%)	Category 2 *n* (%)	Category 3 *n* (%)	
Categories of tumor differentiation patterns	Category 1 (G1, G2)	25 (58.1%)	8 (32%)	N/A	17 (68%)	0.011 *
	Category 2 (G2)	8 (18.6%)	2 (25%)	1 (12.5%)	5 (62.5%)	
	Category 3 (G1, G2, G3)	2 (4.7%)	N/A	N/A	2 (100%)	
	Category 4 (G2, G3)	8 (18.6%)	N/A	4 (50%)	4 (50%)	

G1: well-differentiated tumor; G2: moderately differentiated tumor; G3: poorly differentiated tumor; N/A: Not Available. * A *p*-value < 0.05 is considered statistically significant.

**Table 10:** The relationship between the subcategories derived from Category 3 of CDX2 nuclear expression and tumor differentiation patterns’ categories.

Total Number of Cases Analyzed (*n*) = 43		*n*-Value	Percentage (%)	Chi-Squared Test (*p*-Value)
Subcategories Derived from Category 3 of CDX2 Expression	Categories of Tumor Differentiation Patterns			
**Subcategory 3A**	Total Cases	14	100%	0.0018 *
	Category 1	12	85.7%	
	Category 2	2	14.3%	
**Subcategory 3B**	Total Cases	10	100%	
	Category 1	5	50%	
	Category 2	3	30%	
	Category 3	1	10%	
	Category 4	1	10%	
**Subcategory 3C**	Total Cases	2	100%	
	Category 3	1	50%	
	Category 4	1	50%	
**Subcategory 3D**	Total Cases	2	100%	
	Category 4	2	100%	

* A *p*-value < 0.05 is considered statistically significant.

**Table 11:** Descriptive statistical analysis of the relationship between cases with dMMR/MSI-H phenotype, categories of CDX2 nuclear expression, tumor budding score, and the categories of tumor differentiation patterns.

Oncological Stage	Cases with dMMR/MSI-H Phenotype (Out of 22 Cases Evaluated for the MMR/MSI Status)		Categories of CDX2 Nuclear Expression	Tumor Budding Score	Categories of Tumor Differentiation Patterns
	*n* (%)	*n*			
Stage II	3 (7%)	1	Category 2	BD3	Category 4
		1	Category 2	BD3	Category 4
		1	Category 3	BD2	Category 2

dMMR: Deficient Mismatch Repair; MSI-H: High Microsatellite Instability; MMR: Mismatch Repair; MSI: Microsatellite Instability; BD3: tumor budding score 3 (high score); BD2: tumor budding score 2 (moderate score).

## Data Availability

The original contributions presented in the study are included in the article, further inquiries can be directed to the corresponding authors.
